# Supplementary material for: Telehealth Outreach Program for Child Traumatic Stress: Strategies for Long-Term Sustainability
Source: Healthcare (Basel). 2024 Oct 23;12(21):2110. doi: 10.3390/healthcare12212110 (PMC11545140; doi:10.3390/healthcare12212110)
Supplement: Supplementary file 1 [file healthcare-12-02110-s001.zip › healthcare-3253384-supplementary.pdf]

TF-CBT Clinician Survey

You have participated in a training about telehealth delivery of TF-CBT. We want to ask you about your ongoing experience with telehealth. This survey is anonymous and completing the survey is completely voluntary. If you have any questions, please reach out to the Research Coordinator, Rebecca Verdin, at beeksr@musc.edu.

Please select the date(s) of all the telehealth training sessions you have attended with the MUSC team.

- ☐ 3/5/21
- ☐ 3/24/21
- ☐ 7/15/21
- ☐ 8/5/21
- ☐ 8/6/21
- ☐ 10/7/21
- ☐ 10/13/21
- ☐ 11/5/21
- ☐ 11/19/21

How many clients did you see via telehealth during the 6 months following the first telehealth training?

____

Do you currently see clients via telehealth?

- ◯ Yes
- ◯ No

How many clients are you currently seeing via telehealth?

____

What has helped you continue to use telehealth in your practice?

____

What has made it difficult to use telehealth in your practice?

____

What would have been helpful to support your continued use of telehealth in your practice?

____

If you could wave a magic wand and you, your agency, and your clients could have absolutely any resources, support, etc. to implement telehealth, what would you want?

____
